# A thematic analysis of barriers and facilitators to participant engagement in group exposure and response prevention therapy for obsessive–compulsive disorder

**DOI:** 10.1111/papt.12430

**Published:** 2022-10-27

**Authors:** Tamara Leeuwerik, Giorgia Caradonna, Kate Cavanagh, Elizabeth Forrester, Anna‐Marie Jones, Laura Lea, Claire Rosten, Clara Strauss

**Affiliations:** ^1^ School of Psychology University of Sussex Brighton UK; ^2^ Independent Consultant London UK; ^3^ Sussex Partnership NHS Foundation Trust Brighton UK; ^4^ School of Health Sciences University of Brighton Brighton UK

**Keywords:** barriers, engagement, exposure and response prevention, facilitators, obsessive–compulsive disorder, thematic analysis

## Abstract

Exposure and response prevention (ERP) is the gold standard in the treatment of the obsessive–compulsive disorder (OCD). It can be delivered effectively using an individual or group therapy format. Nonetheless, a sizeable proportion of people diagnosed with OCD do not experience OCD symptom remission following ERP. Research suggests that participant engagement with ERP tasks predicts therapy outcomes but there is little consistent evidence across studies on what predicts engagement. A recent meta‐analysis of participant engagement in cognitive‐behavioral therapy for OCD found that group ERP had a comparatively lower dropout rate than individual ERP. Little is known about participant perceptions of ERP to guide an understanding of how the group therapy format may affect participant engagement. This study conducted a qualitative exploration of what helps or hinders participants' engagement in group ERP. It involved thematic analysis of semi‐structured interview data collected at a 6‐month follow‐up from 15 adults with OCD who took part in group ERP. The study identified five main themes that captured participants' perceived facilitators and barriers to engagement in therapy: ‘Group processes’, ‘Understanding how to overcome OCD’, ‘Personal relevance’, ‘Personal circumstances’, and ‘Attitudes towards ERP’, which captured dynamically inter‐related barriers and facilitators at the level of the client, therapist, therapy and social environment. Each theme and associated sub‐themes are discussed in turn, followed by a consideration of the study's limitations and implications.

**Practitioner Points**

- Delivering ERP as a group treatment can benefit normalisation, provide opportunities for testing obsessive beliefs about intrusions, and promote engagement in between‐ and within‐session ERP by creating a sense of belonging and accountability. However, group composition needs careful consideration; facilitators should acknowledge the heterogeneity of OCD and give due attention to mental compulsions and imaginal exposure.
- In group ERP, non‐attendance can undermine group morale, and this should therefore be addressed openly and proactively. Building 1:1 time into group treatment protocols could provide a vital opportunity to anticipate and respond to clients' reservations or concerns about group ERP.
- Client expectations, motivation, readiness to change, self‐efficacy, perseverance and commitment can fluctuate over the course of therapy. These psychological factors are likely influenced by psychoeducation (through facilitating insight into OCD and a clear understanding of the treatment rationale), personal circumstances, (early) success with ERP, perceived personal relevance of (within‐session) ERP, and, in the context of group ERP, group processes. Therapists should therefore pay close attention to all these factors throughout therapy.
- Clients with co‐occurring depression may require greater between‐session support to facilitate their engagement in ERP.

## INTRODUCTION

This study aimed to explore what participants perceived to be the barriers and facilitators of engagement in group exposure with response prevention treatment for obsessive–compulsive disorder (OCD). OCD is a debilitating mental health condition, characterised by recurring intrusive thoughts, images or urges that cause significant distress and/or repetitive physical behaviours or mental acts aimed at preventing anticipated adverse consequences and alleviating distress (APA, 2013). Exposure and response prevention (ERP), delivered with or without added cognitive therapy (CT) strategies, is the psychological therapy of choice (e.g. APA, 2007; NICE, 2005). It requires clients to engage in repeated, prolonged exposure to obsessions while refraining from compulsions, breaking the vicious cycle of OCD through a process of habituation (Kozak & Foa, 1997) and/or inhibitory learning (Craske et al., 2014).

Treatment typically starts with psychoeducation to inform the rationale for ERP (e.g. Kozak & Foa, 1997), followed by an individually tailored formulation of the person's OCD symptoms. Subsequently, sessions focus on in‐session (in‐vivo and imaginal) exposure to triggers for obsessions. Home practice of ERP is considered a key treatment component to consolidate learning, to ensure clients take responsibility for ERP without the implicit reassurance or safety offered by the therapist's presence and to enable exposure to triggers in their everyday environment (e.g. Franklin et al., 2005).

Exposure and response prevention is the gold standard and a highly effective therapy, yet research suggests that 35% of people diagnosed with OCD experience no discernible change in OCD symptoms following ERP and approximately 50% do not achieve remission (Öst et al., 2015). ERP is anxiety‐provoking by design and often perceived as challenging (e.g. Olatunji et al., 2009). The moderate response rate for ERP might therefore reflect variable client engagement. The latter can be defined as ‘all the efforts that clients make during the course of treatment towards the achievement of changes’ (Holdsworth et al., 2014, p. 430). Client engagement is multifaceted and can be operationalised as: (1) consistent attendance; (2) completing therapy on time; (3) active participation in session; (4) conducting between‐sessions tasks; (5) developing and maintaining an effective client‐therapist relationship and (6) developing and maintaining supportive, helpful interactions with other participants (Tetley et al., 2011).

Within the context of ERP for OCD, relatively little is known about (some of) these different facets of engagement. With regard to consistent attendance and treatment completion on time, a recent meta‐analysis of participant engagement in cognitive‐behavioral therapy (CBT) for OCD found a 16.6% dropout rate for ERP (Leeuwerik et al., 2019). The pooled risk ratio was 2.45 towards early dropout. Some studies have shown that early dropout from ERP is associated with poorer treatment outcomes (e.g. Aderka et al., 2011) so it is important to understand what predicts it. The meta‐analysis found a pooled mean number of 13 attended face‐to‐face sessions of CBT (all types: ERP, CT and a combination of both), in line with treatment duration guidelines (APA, 2007; NICE, 2005). The pooled proportion of attended (face‐to‐face) sessions was 87%, suggesting a relatively high degree of consistent attendance. However, results were based on a small subset of studies, suggesting this aspect of engagement is rarely examined.

When considering active participation in session and conducting between‐session tasks, research into ERP task engagement is too limited and varied to meta‐analyse ERP task completion rates; descriptively, a small body of empirical studies suggests an at least satisfactory average mean level of engagement in home practice (Leeuwerik et al., 2019). Some studies have shown that a high level of engagement in between‐session ERP is required to achieve symptom remission (Abramowitz et al., 2002; Simpson et al., 2011; Wheaton et al., 2016). This supports the central role of home practice in ERP and suggests it is a key aspect of engagement to understand and address.

Relatively little is known about developing and maintaining an effective client‐therapist relationship. A few studies have shown that the working alliance, particularly the extent to which the therapist and client agreed on the tasks of therapy, was positively associated with post‐treatment OCD symptom reduction, through its effect on client adherence to ERP (Simpson et al., 2011; Wheaton et al., 2016). Even less is known about supportive, helpful interactions with other participants and their association with treatment outcomes. Group ERP, delivered with or without cognitive strategies, appears as effective as individual ERP therapy (e.g. Öst et al., 2015) and can increase access to treatment, provided clients feel able to engage in it.

Although Kobak et al. (1995) theorised early on that the group format may facilitate engagement in ERP through normalisation, encouragement, behavioural modelling and social learning, very little is known about how interactions with other participants impact treatment outcomes. Leeuwerik et al. (2019) found a significantly lower pooled dropout rate for group CBT (13%) than individual CBT (17%). However, results were inconclusive as to whether peer support benefitted treatment completion; this warrants further exploration. A recent review of the wider literature on CBT for anxiety disorders (Luong et al., 2020) found just three studies that examined the association of group engagement, i.e. ‘active participation, mutual liking, and collaborative climate within the group (p. 9)’ with (residential, inpatient) cognitive (processing) therapy outcomes, for social phobia (Bonsaksen et al., 2011, 2013) and post‐traumatic stress disorder (PTSD) (Ellis et al., 2014), respectively. Greater group engagement predicted greater (client‐rated) anxiety reduction during (Bonsaksen et al., 2011) and post‐treatment (Ellis et al., 2014) and at one‐year follow‐up (Bonsaksen et al., 2013). Luong et al. (2020) suggest that group engagement may facilitate a sense of cohesion that, in turn, promotes attendance, disclosure and active participation in treatment tasks. The above‐mentioned studies did not explore predictors of group engagement, but Bonsaksen et al. (2011) concluded, from examining the engagement process over time, that sample characteristics (including diagnosis), treatment model and setting were likely influential.

Overall, the extant literature suggests it is important to address client engagement in ERP to benefit treatment outcomes. Yet, despite over 40 years of research into ERP for OCD, relatively little is known about (interactions between) predictors of (facets of) engagement over the course of treatment, to guide this. Quantitative research to date has failed to establish reliable, consistent and robust predictors of participant engagement with ERP therapy to support clinical practice (Knopp et al., 2013; Leeuwerik et al., 2019; Olatunji et al., 2013). Within quantitative research, approaches to identifying predictors of engagement have tended to involve: (i) post‐hoc exploration of variables for which data happens to be available; (ii) brief exit interviews with participants who drop out to understand reasons for non‐engagement; (iii) extrapolating potential predictors of engagement identified in relation to other presenting problems and/or therapeutic approaches and, rarely, (iv) evaluation of potentially important predictors as identified in theories of engagement, e.g. the transtheoretical model of readiness to change (Prochaska & Velicer, 1997; Taylor et al., 2012). What further ails this area of research is a lack of sophisticated measurement of both quantity and quality of engagement with key therapeutic tasks (e.g. Tetley et al., 2011), including in the context of ERP for OCD (Leeuwerik et al., 2019; Wheaton & Chen, 2021). Consequently, ‘remarkably little is known about this issue’ (Taylor et al., 2012, p. 586) and client engagement in psychological therapy is ‘relatively under‐theorised’ (Holdsworth et al., p. 428).

Qualitative research is well‐placed to explore participants' experience of engagement over the course of ERP to inform a greater understanding of potential facilitators and barriers to engaging in ERP and drive a more theoretically informed approach to examining and addressing engagement in ERP (Bryant et al., 1999; Tetley et al., 2011). However, qualitative studies of participants' experience of ERP for OCD are few and far between. A thematic analysis of semi‐structured interviews with eight participants in a group or individual ERP (Lee & Rees, 2011) highlighted they experienced ERP as anxiety‐provoking but that the structured, graded approach to treatment, psychoeducation and combination with cognitive strategies aided engagement. Support from fellow participants, the therapist and family and friends was also important. Personal motivation and courage further helped sustain engagement in ERP. However, this study provided only a brief account of the experience of a few participants in group ERP, lacking more in‐depth information to guide understanding.

Marsden et al. (2018) conducted a comparative thematic analysis of semi‐structured interviews with adults with OCD who completed individual CBT (*n* = 10) and eye movement desensitisation and reprocessing (EMDR) (*n* = 14). General life difficulties such as physical illness, housing and employment issues and practical issues around the timing and location of sessions, impacted participants' ability to get the most out of therapy (CBT and EMDR). Some participants reported that the structure of sessions did not provide enough opportunity to express themselves, to the detriment of the client‐therapist relationship and resulting in dropout. This highlights that specific therapy characteristics have the potential to influence engagement.

Bevan et al. (2010) also provided a comparative thematic analysis of participants' experiences of intensive (*n* = 6) and weekly (*n* = 6) individual CBT, gathered through a semi‐structured interview. Participants commented on the benefits of the intensive format in reducing procrastination and managing anxiety raised by ERP tasks. The intensive format was also experienced as motivating, suggesting that (preference for) therapy format could hinder or facilitate engagement.

## AIMS

Findings from the aforementioned qualitative studies of predominantly individual ERP suggest that a range of individual, interpersonal, therapy‐specific and extra‐therapeutic factors may influence engagement. However, they did not offer a more in‐depth consideration of what may affect (the process of) engagement in group ERP. Therefore, this study aimed to explore what participants perceived to affect their engagement in group ERP for OCD, to contribute to a more targeted approach to improving client engagement in group ERP to benefit outcomes for people with OCD (Barrett et al., 2008).

## METHODS

### Design and procedure

This study involved thematic analysis of semi‐structured interview data collected at a 6‐month follow‐up from 15 adults with OCD who had participated in group ERP. Study data were part of a pilot RCT comparing group ERP (*n* = 18) to mindfulness‐based ERP (MB‐ERP) (*n* = 19) therapy for OCD (Strauss et al., 2015, 2018). A lived experience advisory panel consulted on the development and implementation of the pilot RCT, which received full ethical approval through the South East Coast (Surrey) arm of the National Research Ethics System in the United Kingdom (reference: 13/LO/1768). The pilot RCT was pre‐registered (ISRCTN52684820. Registered on 30 January 2014).

All pilot RCT participants were invited to participate in the interview at a 6‐month follow‐up. The current study reports on the qualitative analysis of the interview data from group ERP participants only. All interviews, ranging from 30 to 60 min, were conducted on NHS premises by a research assistant and were audio‐recorded. The interviews were transcribed verbatim and anonymised by the first author.

### Participants

Within England, improving access to psychological therapy (IAPT) services routinely deliver ERP for OCD in line with National Institute for Health and Care Excellence (NICE) guidelines (2005). NICE (2005) recommend guided self‐help, group and individual ERP interventions as part of a stepped‐care approach to treating OCD.

Participants in the pilot RCT were recruited from IAPT services in an NHS mental health trust in the south of England. The inclusion criteria were: (i) 18 + years of age; (ii) met DSM‐IV diagnostic criteria for OCD (APA, 1994) based on the Mini International Neuropsychiatric Interview [MINI 6.0.0] (Sheehan et al., 2010); (iii) if on psychiatric medication, stable dosage for a minimum of 3 months prior to starting therapy; (iv) no plans to change psychiatric medication during the study and (v) had not received any psychological therapy in the 3 months before the current study, nor planned to engage in psychological therapy during the study. At the time of the study, there were concerns about the suitability/safety of a mindfulness‐based intervention for people with a diagnosis of PTSD (as anecdotal evidence suggested that mindfulness practice could elicit distressing flashbacks and dissociation) or a diagnosis of anorexia (as anecdotal evidence suggested that mindfulness strategies regarding choosing how to respond to behavioural urges could be applied to strengthening to the ability to choose to respond to hunger urges by not eating). The study started shortly after DSM‐5 was published, re‐categorising people with hoarding problems into a separate category distinct from OCD as evidence was increasingly suggesting that hoarding was distinct from OCD in terms of its underlying development and maintenance mechanisms. For these reasons, people meeting diagnostic criteria for these conditions were not included in the study. Exclusion criteria were: (i) identified organic cause for OCD symptoms; (ii) a diagnosed learning disability, psychotic disorder, PTSD, anorexia nervosa, alcohol dependence or substance addiction; (iii) hoarding‐only compulsions (see Strauss et al., 2015, 2018 for further details).

Fifteen of the 18 participants randomly allocated to the ERP condition (with eight participants in one ERP group and seven in another) completed the semi‐structured interview. They all had a diagnosis of OCD at the start of treatment (as above). The mean depression severity of the sample was in the moderate range (*M* = 27.80, *SD* = 14.37), as measured with the Beck Depression Inventory‐Second edition (BDI‐II) (Beck et al., 1996). See Table 1 for further details on sample characteristics.

**TABLE 1:** Sample characteristics (*N* = 15)

Variable	*M* (*SD*)	*N* (%)
Age	32.20 (10.08)	
Age of onset	11.50 (5.06)	
Gender		
Female		8 (53)
Ethnicity		
White British		15 (100)
Education		
Secondary		7 (47)
Higher		8 (53)
Employment		
Employed		5 (33)
Self‐employed		2 (13)
Unemployed		6 (40)
Other (e.g. retired)		2 (13)
Current medication		8 (53)
(some) prior CBT		9 (60)
Dropout	1 (7)	
Sessions attended	7 (3) (range: 1–10)	
ERP tasks completed^a^	24.00 (9.69)	

^a^ Based on 30% returned homework sheets.

### Interview schedule

The semi‐structured Change Interview (Elliott et al., 2001) was used in the pilot RCT to explore the perceived acceptability and benefits of ERP and MB‐ERP as it was designed to ask participants about their experience of psychological intervention. This interview consists of seven main questions that invite interviewees to reflect on therapy change processes (Questions 1 and 2), what they attribute these changes to (Q3), helpful and unhelpful aspects of therapy (Q4 and 6) and factors that they perceive to enable or hinder them in making the most of the therapy (Q5 and 7). It was therefore also well‐suited to the aims of this study.

### Intervention: Group ERP

Treatment involved 10 two‐hour, weekly sessions of group ERP (delivered through two courses). The protocol (Van Noppen, Steketee, & Pato: Group Behaviour Therapy [GBT] Treatment Manual for OCD, unpublished) was adapted to incorporate recommendations for delivering ERP in line with inhibitory learning theory (Abramowitz & Arch, 2014; Arch & Abramowitz, 2015). Two experienced clinical psychologists, one of whom was an accredited CBT therapist with OCD expertise (CS), delivered the treatment. An expert in ERP (EF) provided supervision to both group facilitators. The first session provided psychoeducation about OCD and introduced the rationale for ERP. From session 2 onwards, participants were required to design and conduct within‐ and between‐session ERP tasks (i.e. home practice). Participants were strongly encouraged to complete their planned ERP tasks at least daily between sessions and record this using worksheets. All sessions included reviewing between‐session practice and planning ERP tasks for the following week. Session 10 reviewed the course and invited participants to devise a personalised plan to consolidate learning (see Strauss et al. (2015) for further details).

### Thematic analysis

The Change Interview transcripts were analysed using reflexive thematic analysis (TA) (Braun & Clarke, 2006, 2019), which offers a flexible approach to identifying, analysing and reporting themes within the dataset, allowing for a detailed and thorough description and rich interpretation of the data. Reflexive TA is an organic, open approach to analysing qualitative data that follows six iterative phases (Braun & Clarke, 2006; 2019). All extracts that broadly pertained to client engagement were included in the analysis. Initial codes were applied to any participant statement on what helped or hindered their engagement in group ERP, where engagement was broadly understood to include session attendance and course completion, within‐ and between‐session ERP task engagement, the client‐therapist relationship, and interactions with other participants, i.e. adopting Tetley et al.’s (2014) operationalisation of engagement. An inductive orientation to coding was used and semantic (i.e. descriptive, participant‐driven) codes that captured ‘explicitly stated ideas' (Braun & Clarke, 2019, p. 58) were applied to the data. GC, a clinical psychology master's student, and TL, an experienced clinical psychologist and doctoral researcher, repeatedly read (phase i) and completed the initial coding of two interviews, comparing and discussing their initial coding to enhance the credibility of the analysis (Archibald, 2016). GC completed the initial coding of the remaining transcripts (phase ii) and clustered them into initial (sub‐) themes, capturing both endorsing and disconfirming views across participants (Elliott et al., 1999) (phase iii). Decisions about the relevance and clustering of initial codes into (sub‐ and higher‐order) themes (phase iv) were reached through group consensus (GC, TL and CS) (Harry et al., 2005), ensuring that the interpretation of the data followed from the initial codes and associated extracts (Elliott et al., 1999; Madill et al., 2000). TL completed the final two phases of the analysis, i.e. defining and naming the themes and writing up the analysis. QSR International's NVivo 12 software was used to conduct the analysis.

## RESULTS

Five main themes captured participants' perceived facilitators and barriers to engagement in group ERP (see Table 2). The associated sub‐themes are discussed further below. The theme that was specific to it being a group treatment, i.e. ‘group processes’, is discussed first. This is followed by a discussion of themes that were not necessarily specific to it being a group intervention, even if they cannot be separated from it as participant experiences were within a group context. Pseudonyms were used to protect participant confidentiality.

**TABLE 2:** Main and sub‐themes

Theme	Captures…	Sub‐themes
Group processes	Participants' experiences of being in a group, highlighting group processes that appeared to affect engagement with ERP	(Fear of) opening up Fear of catching other people's OCD
		Belonging and accountability
		(Shared) therapist support
		Continuity
		Individual attention
Understanding how to overcome OCD	Developing an understanding of the cognitive‐behavioural model of OCD, including how ERP targets the maintaining factors in OCD, *how* to conduct ERP and what to expect during ERP	Insight into OCD
		Understanding the treatment rationale
Personal relevance	Whether participants felt that ERP was relevant to their difficulties, related to whether their compulsions were mainly overt or covert	Perceived relevance of ERP for obsessions without overt compulsions
		Perceived relevance of within‐session ERP
Personal circumstances	The impact of mental health problems, other commitments and life transitions, and social support on session attendance and ERP task engagement	Mental health
		Other challenges
		Support from family and friends
Attitudes towards ERP	The ways in which participants perceived that expectations, motivation, readiness to change and commitment and perseverance affected their engagement in ERP	Expectations
		Motivation
		Readiness to change
		Self‐efficacy
		Commitment and perseverance

### Group processes

#### (fear of) opening up

Some participants felt anxious about joining a group and opening up to the other participants, who were ‘total strangers’ (Brian). Participants were not used to talking about their OCD to other people, feared people's reactions, or generally did not feel comfortable in group situations. However, most participants tolerated their anxiety, e.g. by reminding themselves it was a recommended treatment, and it generally decreased quickly once they got to know the other group members.

#### Fear of ‘catching’ other people's OCD’

Hearing about other people's OCD symptoms caused some participants some initial concerns about ‘catching other people's OCD’(Charlotte) and contributed to Sadie dropping out because: ‘I didn't think I was strong enough to handle the worry of taking on everyone else's situation’. Oscar expressed ambivalence about hearing others talk about recognisable difficulties, as it ‘is sometimes much more acutely distressing than it is valuable’. Mostly, however, participants highly valued sharing experiences and feeling listened to and understood; it helped them to normalise their own intrusive thoughts and made them feel less alone.

#### Belonging and accountability

Some participants described developing a sense of belonging, ‘feeling part of the group’ (Alex), and accountability that motivated them to attend sessions. Anticipating group discussion about home practice acted ‘like a prompt’ (Alex) to do between‐session ERP whilst the sense of accountability also facilitated within‐session ERP, as described by Luke ‘you think ‘well I've got to do something to sort of keep sort of on the same level as everyone”. Seeing other participants struggling to engage with ERP motivated some participants to keep on track, e.g. ‘It makes you think to yourself ‘well you did not do it either, then help yourself out” (Charlotte).

#### (shared) therapist support

Therapist support included practical assistance and emotional support, e.g. helping participants to correctly conduct and persist with ERP tasks, talking them through their home practice, providing encouragement and praise. Participants positively reflected on the therapists' expertise, understanding, warmth or ‘pastoral care’ (Oscar), which helped them to remain engaged, e.g. ‘…they were so understanding […] that really helped ‘cos otherwise I wouldn't have come back’ (Charlotte). Knowing that therapists were there to support them made some participants feel less alone in dealing with OCD. Participants perceived that therapists moved at the group's pace, showing an interest in their feelings and doubts, adjusting sessions to their needs. Discussing home practice and praising achievements increased participants’ confidence in their ability to conduct ERP independently. Moreover, therapists reflected on shared difficulties in conducting ERP, which enabled the group to come up with solutions. However, a couple of participants lacked the confidence or inclination to ask the facilitators for help, which affected their ability to engage in ERP.

#### Continuity

Non‐attendance of group members resulted in different group configurations for some sessions, which some participants found frustrating, disappointing or difficult to understand: ‘It's like, ‘If me, you, you and you is doing it then why is no‐one else?” (Brian). One participant lamented that this resulted in some repetition of course content. However, some participants felt that a smaller number of attendees allowed more time to talk and receive individual attention from the therapists.

#### Individual attention

Some participants found the relative lack of individual attention from the therapists, resulting from the group format, challenging. They would have liked some time alone with the therapists to discuss problems that were difficult to share in a group, making therapists aware of important personal circumstances or feeling reassured about their difficulties. This theme reflected that not all participants felt comfortable talking in the group (‘opening up’ sub‐theme) or did not wish to take up time to discuss individual difficulties. However, some participants felt there was still ample scope for individually tailored therapy, appreciating ‘…the ability of the staff to react to your specific needs’ (Oscar).

### Understanding how to overcome OCD

#### Insight into OCD

Some participants stated that psychoeducation about OCD, particularly how avoidance and compulsions maintained OCD, changed their perspective on their symptoms and helped them gain the resolve to engage with ERP tasks, as illustrated by Michelle: ‘I'm just not doing it[ritual] and that does feel better, I still feel a little bit guilty but I'm trying to make myself realise that I'm not achieving anything by doing that [the rituals], it's just the OCD taking over ’.

The group context may have helped facilitate such insight, e.g. Charlotte described: “It's been helpful to look at the OCD model and fit myself into it. It's also been, also helpful then to see other people being fit in alongside it even though they've got very different issues, to see how it all fits in the same model.”

### Understanding the treatment rationale

Most participants found that learning how and why ERP works, including psychoeducation about the (self‐limiting) fight‐flight response, motivated them to engage in ERP, e.g. ‘… it […] made me want to tackle it instead of dwelling on it’ (Helen), and increased their self‐efficacy‘[…] you can be like ‘oh I can do this thing that I find really scary and it won't always be scary”(Isabel). Psychoeducation was revisited throughout therapy, which helped participants get to grips with ERP: ‘…it took quite a long time to actually grasp what I needed to do and why’ (Charlotte).

### Personal relevance

#### Perceived relevance of ERP for obsessions without overt compulsions

Participants with obsessions without overt compulsions experienced challenges in successfully conducting (imaginal) ERP tasks: ‘…a lot of people suffer inside their heads, so that was difficult for them [the facilitators], to make a situation relevant’ (Adam). This created a sense that there was not a good fit between their symptoms and the therapy: ‘I didn't really fit into their system of exposure’ (Chloe). Several participants described trial and error in successfully triggering intrusive thoughts: ‘[…] they suggested things, and I tried it and they weren't really working for me so I had to sort of keep trying different things’ (Luke). These participants also expressed some disappointment that the course gave mental compulsions relatively less consideration.

#### Perceived relevance of within‐session ERP

Some participants experienced difficulties with within‐session ERP tasks because their OCD symptoms predominantly occurred within their home environment. This contributed to their perception that engaging in within‐session ERP tasks was not particularly relevant, e.g. ‘it wouldn't do anything for me’ (Adrian). For others, the artificiality of intentionally provoking their obsessional intrusions in the session rendered it ineffective: ‘[…] sometimes trying to provoke that anxiety, because I knew I was doing it for that reason, was quite tricky’ (Jessica).

### Personal circumstances

#### Mental health

Some participants experienced depression and anxiety and/or severe OCD symptoms, which impacted their ability to confront OCD, e.g. Alex stated he ‘couldn't summon the kind of strength to … take the illness on’. It contributed to Adam missing sessions: ‘I was disappointed with myself that I didn't go as much as I wanted to…maybe ‘cos I do suffer with depression’. For some participants, OCD or co‐occurring depression and/or anxiety symptom severity did not affect their attendance but hampered their ability to engage with between‐session ERP tasks, e.g. ‘The anxiety mixed with like depression, made it kind of difficult [to prevent rituals]’ (Chloe).

#### Other challenges

For participants with children, difficulties securing childcare contributed to missing sessions. Other factors, such as the clinic's central location and job flexibility made it easier for some participants than others to attend (day‐time) sessions. Life commitments made it hard to prioritise between‐session ERP tasks for some.

Some participants experienced stressful life events and instability, e.g. moving to a new house, which affected their engagement. Conversely, suitable accommodation, e.g. having privacy, facilitated engagement in between‐session ERP tasks for others. For some participants, life transitions preoccupied them and made them question the timing of the course ‘…it's quite a bad time to like do anything’ (Isabel).

#### Support from family and friends

Family support with childcare enabled participants with children to attend sessions. Loved ones also encouraged participants to continue to attend sessions, or as Charlotte described: ‘helping me to find the wherewithal to go to the group each week’. Some participants found it helpful to ask family members to remind them to do their ERP tasks.

### Attitudes towards ERP

#### Expectations

Participants typically discussed their expectations when asked the questions of what personal resources or limitations may have made it easier or harder to make use of the therapy; they perceived their expectations to influence their ability to make use of the therapy. For example, Freddie commented ‘I'm actually in general a quite lazy person so I'm quite surprised that I actually did any of the tasks …maybe it was like a belief that it could, like, improve.’ Several participants longed to be rid of OCD altogether, often coupled with an appreciation this was not necessarily realistic: ‘I have an illusion that you can be completely fine afterwards and not have it affect you at all, but I think that's obviously not the case’ (Isabel) as OCD can be a ‘life‐long battle’ (Brian), and ‘it's more about learning how to cope with it’ (Charlotte). As therapy progressed, hopes of getting better typically increased but some participants described that this fluctuated: […] there are always times when you are uncertain if it's the right thing’ (Isabel). Therapists played an important role in helping participants to know what to expect, e.g. that starting ERP was going to be challenging. Therapists managed expectations about remission, which caused Charlotte some initial anxiety: ‘I suddenly thought: ‘Oh my God, I might not be fine, I might not be cured”. She subsequently felt that having a more realistic perspective helped to galvanise her:‘My new goal was to understand what we were trying to achieve and to be able to take things away to help myself afterwards'.

#### Motivation

Many participants described an inner drive or determination to face OCD head‐on and engage in ERP tasks. Many participants felt they owed it to themselves to succeed and no longer wanted the OCD to take over their lives. Other participants were primarily motivated to engage with therapy for the sake of their children and families, wanting to set a good example, make their family proud or reduce the impact that OCD had on them, e.g. ‘I'd like to show that I can change and I can make life easier’ (Michelle).

#### Readiness to change

Most participants talked about whether they felt ready to change, e.g. ‘I'm at a point in my life when I thought ‘Enough is enough, I'm ready to receive help” (Charlotte), with some expressing they had no other choice than ‘facing things head on’, e.g. ‘I was just at the end of my tether […] I had to do it’ (Adam). Several participants with very longstanding OCD symptoms wanted more sessions to settle into treatment and fully engage in between‐session ERP: ‘[…] with the knowledge of this session every week, I think in time […] I would have got better at that’ (Alex).

#### Self‐efficacy

Many participants expressed that a sense of self‐efficacy contributed to their engagement in therapy and vice versa: ‘I think my confidence and being able to do things has gone up […] realising it [ERP] is actually not that bad’ (Luke), ‘Some things you thought were impossible … you can sort of do them’ (Derrick). Other participants understood the rationale for ERP but lacked confidence in their ability to do it: ‘I don't really believe I can follow it’ (Oscar).

#### Commitment and perseverance

Commitment and perseverance were considered important attributes in tolerating the distress associated with ERP, particularly when motivation was at a low ebb: ‘Having the willpower, you know you don't like it, if you don't have that, it would be a very difficult thing’ (Derrick). This was also related to a belief that ERP would work and make them feel better, i.e. the credibility of the treatment, e.g. ‘…when I really did not want to do the tasks […] knowing that if you do these things, things can get better, has made me sort of push through when things seemed a bit hard’ (Luke).

## DISCUSSION

This qualitative study examined participant perspectives on facilitators and barriers to engagement in group ERP for OCD. The five themes related to consistent attendance, effective working relationships with other participants and the therapist, and within‐ and between‐session participation in ERP (Tetley et al., 2011). Perceived barriers and facilitators to engagement in group ERP appeared to broadly come under group therapy (the ‘group processes’ theme), general ERP therapy (e.g. ‘understanding how to overcome OCD’, ‘personal relevance’; 'attitudes towards ERP') and extra‐therapy factors (‘personal circumstances’).

The group‐specific theme, i.e. group processes, highlighted that most participants described a sense of belonging and accountability to the group, reflecting effective working relationships with other participants. ‘Fear of opening up’ suggested participants' confidence in social situations and (feelings around) disclosure of OCD symptoms (initially) affected their ability to develop good working relationships with each other. The quality and availability of (individualised) therapist support, and (participants' reactions to the) continuity of the group also had the potential to affect engagement with the group and with ERP. Participants in Lee and Rees (2011) similarly highlighted that being in a group was normalising and encouraging and that therapist support benefitted participants' engagement in ERP. Participant reservations about being in a group were also touched on by eligible participants in a trial comparing individual versus group therapy who refused group ERP based on general social anxiety, fears about catching other people's OCD symptoms, lack of personal attention and/or a sense of shame about their OCD symptoms (O'Connor et al., 2005). Overall, group processes appeared to create a unique group ‘climate’ (Tasca et al., 2006; Paquin & Kivlighan, 2016) or ‘cohesion’ (e.g. Burlingame et al., 2018; Kobak et al., 1995) that could promote or inhibit engagement in ERP. While a few studies of individual ERP have demonstrated that the client‐therapist working alliance can affect treatment outcomes through its effect on ERP task engagement, this study suggests the quality of working relationships between participants may also affect outcomes through its impact on participants' ability to ‘do the work required in therapy’ (Tetley et al., 2011, p. 928).

A general ERP therapy factor implicated in the themes ‘insight into OCD’ and ‘understanding the treatment rationale’ was the extent to which psychoeducation about OCD and the rationale for ERP resonated with participants and made the therapy seem credible. A few quantitative studies of engagement have found that the pre‐treatment degree of insight (De Araujo et al., 1996; Simpson et al., 2011; Tolin et al., 2004) affects ERP task engagement. By extension, improving insight through psychoeducation is likely to positively affect engagement. Psychoeducation is tied up with therapist expertise and quality of delivery and bears the imprint of the client‐therapist working alliance. In line with current study findings, a recent review of research into homework completion for ERP (with adults or children) (Wheaton & Chen, 2021) concluded, based on a small number of studies, that participant understanding of the ERP rationale and agreement with the therapist on therapy tasks appeared to predict better homework completion.

The perceived personal relevance of (within‐session) ERP also impacted participants' capacity to engage in it and appeared to affect their sense of belonging, ability to share and compare experiences and their hopes and expectation of improvement. Findings also suggested that participants with mental rather than overt compulsions experienced ERP as less relevant and/or effective, which affected their engagement. This touched on the fact that OCD characterised by mental compulsions may be more difficult to treat with (imaginal) ERP than overt compulsions (Clark & Purdon, 1993; Steketee et al., 2019; Whittal et al., 2010).

The ‘attitudes to ERP’ (sub‐)theme(s) resonate with theoretical models of behaviour change (e.g. Prochaska & Velicer, 1997), studies of participant engagement in psychological therapy in general (e.g. Holdsworth et al., 2014) and a few quantitative studies of ERP showing that motivation (e.g. Bachofen et al., 1999), readiness for change and treatment expectations (see Wheaton & Chen, 2021), predicted (between‐session) ERP practice.

Some themes captured extra‐therapy factors, e.g. they reflected that client characteristics such as the nature and severity of (co‐occurring) mental health difficulties may affect engagement. This is supported by anecdotal evidence suggesting OCD symptom severity and co‐occurring depression may be associated with poor outcomes (e.g. APA, 2013; Foa et al., 1999; Salkovskis & Westbrook, 1989) and a systematic review showing a reasonably consistent pattern of a positive association between severity of OCD, anxiety and depression and drop‐out status (Knopp et al., 2013). In this study, however, participants perceived that (co‐occurring) symptom severity did not necessarily affect dropout or session attendance but affected their ability to engage in between‐session ERP tasks, in accordance with a review by Holdsworth et al. (2014) showing that within‐session engagement did not necessarily correspond with between‐session engagement. However, Wheaton and Chen's (2021) review concluded that research was equivocal as to whether OCD symptom severity at baseline predicted homework completion; this warrants further exploration.

Participants' social context, including stressful life events and changes, job flexibility, childcare commitments and access to social support, also seemed to affect session attendance and between‐session ERP task engagement, including through its impact on readiness for change, motivation, commitment and perseverance. Marsden et al. (2018) similarly reported that general life problems affected engagement among CBT participants.

There appeared to be many reciprocal relationships between (sub‐)themes. For example, ‘Understanding how to overcome OCD’ appeared to impact participants' motivation to initiate and persevere with ERP. Psychoeducation may affect motivation through increasing insight into OCD and understanding of the treatment rationale, as well as affecting self‐efficacy and expectations about therapy. Motivation was bolstered by support from therapists, loved ones and a sense of accountability and belonging to the group. Participant accounts in Marsden et al. (2018) similarly suggested that the therapeutic alliance influenced motivation, including by creating positive treatment expectations through (persuasive) psychoeducation about the treatment rationale. Participant expectations, readiness to change and self‐efficacy all seemed to fuel motivation whilst commitment and perseverance, when motivation was low, was influenced by self‐efficacy and the perceived relevance, or suitability, of the therapy. This resonates with theoretical perspectives that propose motivation is shaped by treatment expectations and self‐efficacy (Drieschner et al., 2004). Participants also described a reciprocal relationship between early success with ERP and therapy engagement, seemingly through increasing motivation, perceived self‐efficacy and the credibility of the therapy. Overall, study findings support the notion that motivation is ‘a dynamic treatment target to enhance participation’ (Holdsworth et al., 2014, p. 435).

### Limitations

Participants were White British and were recruited through two UK NHS IAPT services in a mental health trust in the South of England, which limits the transferability of results. Participants took part in a pilot RCT and all but one completed therapy, suggesting they were perhaps more motivated than participants in routine group ERP. Nonetheless, their accounts reflected struggles with engagement that are likely to resonate with adults who seek routine treatment for OCD. It is not inconceivable that those who chose not to participate in the interviews (at follow‐up) were the least engaged in the intervention or otherwise represented a unique subset of participants whose perspectives on engagement were not included.

The interviews were conducted by someone independent of therapy delivery but were nonetheless potentially subject to social desirability bias. The Change Interview invites participant reflection on (un)helpful aspects of treatment and personal characteristics and circumstances that may influence engagement, but does not exhaustively probe facets of engagement. Furthermore, the interviews were held at a 6‐month follow‐up; participants' accounts may have been influenced by whether they experienced sustained therapeutic gain. The time frame may also have affected recall of their therapy experience, although it may have also allowed consolidation of learning and understanding of facilitators and barriers to engagement.

The study explored engagement within the context of group ERP and evidently does not necessarily elucidate what might affect engagement in individual ERP, although the findings resonated with qualitative evaluations of individual ERP (combined with CT).

### Research implications

The therapeutic alliance has been researched widely as a common factor of therapeutic change (e.g. Wampold, 2015). However, the interactions and relationships between participants are not normally the focal point of therapeutic exploration and change in group ERP, yet clearly can influence engagement in ERP and warrant further research. Capturing ‘live’ engagement processes, e.g. through recording sessions, would allow exploration of how the group climate affects engagement (Elliott, 2010; Rhodes, 2011).

Whilst some of the study themes were specific to ERP, others were reflective of group therapy, general therapy, and extra‐therapy factors, albeit influenced by ERP therapy characteristics; e.g. accountability to the group was informed by the fact that therapy required both within‐ and between‐session ERP. Whether client engagement in ERP is aided or hindered by specific therapeutic techniques and the nature of OCD symptoms or more likely to reflect common, trans‐diagnostic and trans‐therapeutic factors warrants further investigation (Wampold, 2015). The dynamic interplay between group therapy, ERP, general therapy and extra‐therapy factors that affect engagement in group ERP could be examined further by developing a grounded theory of the process of patient engagement in group ERP (Corbin & Strauss, 2015). Quantitative research involving psychometrically robust and repeated measurement of ERP practice and potential predictors of engagement identified in this study (Holdsworth et al., 2014) could similarly elucidate the engagement process and its relationship with therapy outcomes.

The results could also inform the development of a measure of client engagement for research or clinical purposes, e.g. to measure engagement across therapy. Findings also highlight the importance of taking account of OCD symptoms subtypes when examining engagement.

Further exploration of the themes from this study with people who dropped out from group ERP is also likely to advance understanding of what hinders participant engagement. The development of a semi‐structured interview specifically designed to probe the various aspects of participant engagement would also benefit research in this area.

### Clinical implications

Group composition and group cohesion need careful consideration as they may get in the way of engagement in ERP. This need to be openly addressed, e.g. therapists should anticipate and discuss anxiety clients may experience about being in a group; disclosing OCD symptoms to the group may be particularly challenging for clients with ‘forbidden’ thoughts (e.g. Kobak et al., 1995). Non‐attendance could impact on remaining participants (Paquin & Kivlighan, 2016) and must be addressed proactively. The extent to which participants achieve success with ERP tasks could also affect the morale of the group.

As within‐session ERP was not necessarily experienced as effective, home treatment should be considered when required (e.g. Rowa et al., 2007), taking into account the additional resource demands in publicly funded health care services. Therapists need to create a positive but realistic expectancy of the efficacy of therapy, aided by appropriate psychoeducation. Clients with co‐occurring depression may require more between‐session support, e.g. between‐session phone calls or home visits, to support their engagement in ERP.

## AUTHOR CONTRIBUTION

CS, LL, CR, AMJ and EF designed the study. GC and TL conducted the thematic analysis. TL wrote and revised the manuscript, under the supervision of CS and KC. All authors contributed to and have approved the final manuscript.

## CONFLICT OF INTEREST

All authors declare that they have no conflict of interest.

## Data Availability

The data that support the findings of this study are available on request from the corresponding author for ethically approved research purposes. The data are not publicly available due to privacy or ethical restrictions.
